# Complete Genome Sequences of Three Bacillus amyloliquefaciens Strains That Inhibit the Growth of Listeria monocytogenes *In Vitro*

**DOI:** 10.1128/genomeA.00579-18

**Published:** 2018-06-21

**Authors:** Thao D. Tran, Steven Huynh, Craig T. Parker, Robert Hnasko, Lisa Gorski, Jeffery A. McGarvey

**Affiliations:** a Department of Plant Sciences, University of California, Davis, California, USA; b Produce Safety and Microbiology Research Unit, Agricultural Research Service, U.S. Department of Agriculture, Albany, California, USA; c Foodborne Toxin Detection and Prevention Research Unit, Agricultural Research Service, U.S. Department of Agriculture, Albany, California, USA

## Abstract

Here, we report the complete genome sequences of three Bacillus amyloliquefaciens strains isolated from alfalfa, almond drupes, and grapes that inhibited the growth of Listeria monocytogenes strain 2011L-2857 *in vitro*. We also report multiple gene clusters encoding secondary metabolites that may be responsible for the growth inhibition of L. monocytogenes.

## GENOME ANNOUNCEMENT

Bacillus amyloliquefaciens is a Gram-positive, motile, rod-shaped bacterium that is commonly found in soil (1). Some strains of B. amyloliquefaciens have been shown to promote plant growth and are used commercially as biofertilizers and biocontrol agents against plant pathogens (2). B. amyloliquefaciens strains ALB65, ALB69, and ALB79 were isolated from alfalfa silage, almond drupes, and grapes, respectively, grown in northern California. These strains have been screened and characterized *in vitro* for the ability to inhibit the growth of Listeria monocytogenes strain 2011L-2857, the etiological agent of a fatal cantaloupe-associated multistate outbreak in the United States (3).

To elucidate the nature of the biocontrol mechanisms of these strains, their whole genomes were sequenced with Pacific Biosciences (PacBio) RS II and Illumina MiSeq platforms. The PacBio reads were assembled using the RS hierarchical genome assembly process (HGAP) version 3.0 in SMRT Analysis version 2.2.0 (Pacific Biosciences, Menlo Park, CA). Single nucleotide polymorphisms (SNPs) within the PacBio contigs were validated by Illumina MiSeq reads using the Geneious software (v11.1.3) (Biomatters, Ltd., Auckland, New Zealand). The NCBI Prokaryotic Genome Automatic Annotation Pipeline (PGAAP) (https://www.ncbi.nlm.nih.gov/genomes/static/Pipeline.html) was used to annotate protein-, rRNA-, and tRNA-coding genes with additional manual annotation based on the genome of Bacillus amyloliquefaciens DSM 7 and its *dnaA* gene (GenBank accession number FN597644).

The chromosomes for strains ALB65, ALB69, and ALB79 were 4,041,665 bp (211.33× mean coverage; GC content, 46.4%), 4,046,611 bp (231.78× mean coverage; GC content, 46.5%), and 3,982,905 bp (304.95× mean coverage; GC content, 46.4%), respectively. The ALB65 genome is predicted to have 3,913 coding sequences (CDS), 9 rRNA operons, and 86 tRNAs. The ALB69 chromosome is predicted to carry 3,911 CDS, 9 rRNA operons, and 86 tRNAs. The ALB79 chromosome is predicted to possess 3,807 CDS, 9 rRNA operons, and 87 tRNAs. All strains harbored prophage sequences, as indicated by PHASTER (http://phaster.ca) (4, 5). B. amyloliquefaciens ALB65 was found to contain 2 questionable and 2 incomplete prophage sequences in its chromosome. The chromosome of ALB69 harbored 1 intact, 1 questionable, and 1 incomplete prophage, whereas ALB79 contained 1 questionable and 1 incomplete prophage sequence in its chromosome. The presence of insertion sequences (IS) was determined using ISfinder (https://www-is.biotoul.fr/) (6), and 54 IS were found in ALB65, 46 IS in ALB69, and 49 IS in ALB79. *In silico* analysis of the genomes via antiSMASH (7–9) revealed that the genomes of ALB65, ALB69, and ALB79 harbor multiple gene clusters predicted to encode for the biosynthesis of bacteriocins and terpenes; nonribosomal peptide synthetases (NRPS) for the production of surfactin, fengycin, bacillibactin, and bacillaene; and the antibiotics difficidin, bacilysin, and macrolactin. These secondary metabolites possess antiviral, antibacterial, and antifungal activities (10) and may be responsible for the growth inhibition of L. monocytogenes by these strains.

### Accession number(s).

The whole-genome sequences have been deposited in DDBJ/ENA/GenBank under the following accession numbers: for strain ALB65, CP029069 (BioProject number PRJNA453418, BioSample number SAMN08978284), for strain ALB69, CP029070 (BioProject number PRJNA453623, BioSample number SAMN08984034), and for strain ALB79, CP029071 (BioProject number PRJNA453625, BioSample number SAMN08984036).
